# Using the Researcher Investment Tool to inform a clinical and translational research initiative

**DOI:** 10.1017/cts.2025.10125

**Published:** 2025-09-12

**Authors:** Brenda M. Joly, Kassandra A. Cousineau, Carolyn E. Gray, Valerie S. Harder

**Affiliations:** 1 Public Health Program, Muskie School of Public Service, University of Southern Maine, Portland, ME, USA; 2 Department of Pediatrics and Vermont Child Health Improvement Program, The Robert Larner M.D. College of Medicine, University of Vermont, Burlington, VT, USA; 3 Catherine Cutler Institute, Muskie School of Public Service, University of Southern Maine, Portland, ME, USA

**Keywords:** Research career progression, data collection tool, measurement instrument, data collection, clinical and translational research evaluation

## Abstract

**Background:**

Numerous efforts are focused on building the clinical and translational research (CTR) workforce. Approaches to evaluate CTR initiatives are varied, and efforts often rely on research project-level outcomes. This article applies an evaluation tool to capture individual-level data.

**Objective:**

The study used a novel Researcher Investment Tool (RIT) to measure researchers’ experience as well as perceptions of institutional support, including an analysis based on researcher characteristics. The study also evaluated the RIT based on common measures, including a bibliometric indicator, investigator status, and percent time dedicated to research.

**Methods:**

The RIT was administered to researchers who received funding or targeted research support from a CTR initiative. Mean scores were assessed by RIT section, domains/sub-domains, and for each item. Mean scores per section were compared across researcher characteristics using t-tests, and associations between common measures and average domain scores were tested using linear regression.

**Results:**

Thirty researchers completed all RIT items. RIT domain scores ranged from a high mean of 4.0 for the research skills domain to a low mean of 2.6 for researcher productivity and community engagement domains. Analysis of indicators of commonly used measures across domains suggest that researchers with a higher bibliometric score had more advanced research skills, service to profession, research productivity, and research collaboration (*p* < .05). New investigators had lower perceptions of institutional support (*p* < .05).

**Conclusions:**

As an evaluation tool, the RIT captures individual-level data that may help to determine key areas of strength and opportunities for growth of a CTR program.

## Introduction

The need for a strong and diverse research workforce to address health issues is well documented in the literature [1–9]. The National Institutes of Health (NIH) has a congressionally mandated program known as the Institutional Development Award (IDeA) [10]. The purpose of the program is to build research capacity in states with historically low levels of NIH funding by strengthening the ability of researchers to conduct and successfully compete for clinical and translational (CTR) research funding [10]. The IDeA CTR Network award program currently funds 11 statewide or multi-state networks in the United States to build biomedical research capacity. A key aim of this federal program is to support the career trajectory of new and early-stage clinical and translational researchers.

One recipient of this IDeA funding was the Northern New England Clinical and Translational Research (NNE-CTR) Network. The NNE-CTR was launched in 2017 and received a second cycle of competitive funding with an overarching goal of fostering research to address health challenges in Maine, New Hampshire, and Vermont [11]. To date, the NNE-CTR has funded 46 small research projects. The NNE-CTR has also provided research supports such as mentorship, professional development, research navigation, community engagement and rural health navigation, access to laboratory testing, and more, to help investigators build skills, enhance their confidence, and gain experience with the research process.

Despite the focus on building and evaluating the health research workforce through efforts such as the NNE-CTR, there are limited tools and systematic methods available to assess how a researcher’s experiences change over time and how their career progresses, especially as systems of support are established to promote research. Tracking individual-level changes remains a key objective of initiatives such as the NNE-CTR, yet there is a gap in comprehensive, broadly applicable tools that offer standardized and consistent measurement [12–14]. Despite several theoretical approaches, metrics, and evaluation tools for assessing CTR initiatives [15–30], there are few validated survey instruments that address the full spectrum of a researcher’s experience across multiple domains. Many of the existing efforts focus on traditional research productivity measures related to quantifying publications, tracking bibliometric indicators, and calculating the number, size, and type of research grants [12]. Recently, studies have focused on the widely used H-index [31–33] as well as exploring demographic and employment characteristics (e.g., early-stage researchers, protected research time) [3,34–36]. A review of published measurement tools revealed survey instruments that were often narrowly tailored to specific aspects (e.g., mentorship, impact), as well as approaches that focused on assessing the attributes, activities, and skills of a researcher [12].

**Figure 1.**
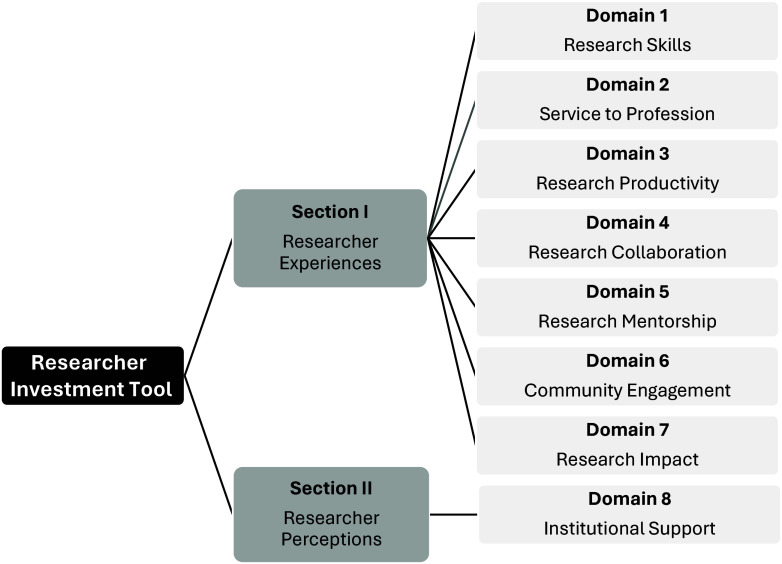
Researcher Investment Tool sections and domains.

In response to the measurement gaps, the Researcher Investment Tool (RIT) was developed to more fully capture broadly defined research domains relevant to the CTR, thus offering a more comprehensive approach. As seen in Figure 1, the tool includes traditional productivity measures and research skills and draws from many of the existing tools that are narrowly focused on one area. In a single tool, the RIT broadly captures researcher perceptions of institutional support and their experiences related to research service activities, collaboration, mentorship, community engagement, as well as the overall impact of their research. The RIT spans eight domains and it offers a standardized approach that can be used over time to measure changes. As previously reported, initial psychometric testing of the RIT revealed good reliability and validity, suggesting its application is both consistent and accurate [14].

The primary objective of this study was to apply the RIT to a sample of NNE-CTR researchers to determine their experiences and perceptions overall and based on researcher characteristics. In addition, a secondary objective was to further explore the validity of our tool by testing associations between individual research domain scores based on three commonly used indicators [31–39] to further evaluate the tool’s applicability including: (1) a widely used bibliometric known as the H-Index score [31], (2) NIH investigator status [37,38], and (3) the percent time dedicated to research [36,39]. We anticipated higher RIT domain scores among those with a higher H-Index score, and lower scores among new or early-stage investigators as well as those who had 50% time or less designated to research.

## Methods

### Sample

A total of 48 NNE-CTR Network members were invited to participate. Inclusion criteria involved receiving small research project funding, serving as a research clinical scholar, or participating in a “research studio” that offered individualized and intensive research support. This group was selected based on the targeted and ongoing wrap-around research supports they received through the NNE-CTR. The RIT was administered in May of 2024, via Qualtrics, an online survey platform. The survey took approximately 15 minutes to complete, and included items that operationalized all eight domains based on a five-point Likert-type scale. The first seven domains assessed self-reported level of experience (1 = no experience, 5 = extensive experience) and the eighth domain measured self-reported perceptions regarding institutional support (1 = not at all, 5 = to a great extent). The survey also included a demographic section to capture the characteristics of researchers. Respondents had five weeks to complete the survey, and reminder emails were sent to non-respondents each week.

### Researcher Investment Tool (RIT)

The RIT is a standardized and validated approach for capturing individual-level measures across eight research domains related to CTR. The survey tool was uniformly developed based on an extensive review of the literature; it follows a consistent structure and format for application, and it has demonstrated consistency over time. As noted above, psychometric testing revealed strong content validity, acceptable internal consistency for all domains, and strong test–retest reliability for nearly all domains [14]. The RIT has 90-items to assess experiences and perceptions of researchers (Figure 1). The first section measures researcher experiences based on 79 items across seven domains, and four sub-domains. The domains focus on research skills, service to the profession, productivity, collaboration, mentorship, community engagement, and research impact. Participants were asked to rate their level of experience using a five-point Likert-type scale anchored by 1 “No Experience” and 5 “Extensive Experience.” The second section measured researcher perceptions based on 11 items assessing one domain known as institutional support. In this second section, participants were asked to respond to a 5-point Likert-type scale anchored by 1 “Not at all” and 5 “To a great extent.” Participants’ RIT mean scores for each domain were calculated by adding the Likert scores within a domain and dividing by the number of items in the domain. Participants’ mean scores for each sub-domain and section were calculated the same way.

### Demographic measures and common indicators

Participants were also asked about demographics regarding their gender (male, female, prefer not to answer), race (White, non-White), ethnicity (Hispanic, non-Hispanic), highest academic degree (MD/DO, all other degrees), and provision of clinical care (yes, no). Additionally, participants were asked about their research careers, which included research specialty (categorized as clinical research, other non-clinical research) and NIH investigator status (new investigator, early-stage investigator) with the answer options “yes,” “no,” or “not sure” for both items. New investigator was defined as “someone who has not competed successfully for a substantial research grant from NIH.” Early-stage investigator status was defined as “someone who has completed their terminal research degree or end of post-graduate clinical training within the past 10 years and who has not previously competed successfully as a PD/PI for a substantial NIH independent research award.” Participants were asked, “In an average month, what percent of time do you spend doing research”?, and answer options included “none,” “1%–10%,” “11%–25%,” “26%–50%,” and “greater than 50%.” Finally, we included the H-Index; a widely used bibliometric score that measures the productivity and citation impact of a researcher’s published work [14]. The H-Index is among the most cited bibliometric measures of research output among health services researchers [12]. We identified and recorded the H-Index score for each participant first through Google Scholar, an approach published by others [40]. If there was no profile, we reviewed ResearchGate, then Scopus to identify individual scores. For our secondary objective, we focused on four common indicators that have been previously studied [31–36] including the H-Index, NIH new investigator status, NIH early-stage investigator status, and percent dedicated time for research.

### Data analysis

Data were cleaned and analyzed using statistical software package Stata 17. In order to address objective one, we focused on scores, overall and based on researcher characteristics. We ran frequency analyses to calculate the maximum, mean, standard deviation, and range for each section, domain, sub-domain, and item. We tested associations between participant characteristics and mean scores for researcher experience (section one), and researcher perceptions (section two) using independent *t*-tests. Research specialty was dichotomized into clinical research versus all other specialties. Percent dedicated time for research was also dichotomized into less than or equal to 50% time and greater than 50% time dedicated to research. Participants indicating gender as “prefer not to say” (*n* = 2) and “not sure” as NIH early-stage investigator status (*n* = 3) were dropped from these analyses. Analyses on race/ethnicity were not possible due to low variability. We compared each total domain score with the maximum potential score to calculate the percent of each domain score achieved.

In order to address the second objective, we used simple linear regressions to test associations between mean item scores within each of the eight RIT domains and our list of common indicators used in prior research. These metrics included H-Index score (continuous), NIH new investigator status (yes/no), NIH early-stage investigator status (yes/no), and greater than 50% time dedicated to research (yes/no). For all statistical tests, we used a *p*-value cutoff of 0.05 to indicate statistical significance.

## Results

Of the 48 invited, 30 participants completed the RIT and demographic questions, for a response rate of 63%. All participants answered each item on the RIT. One participant did not respond to three of the four demographic questions related to research focus. All other demographic questions were fully completed by all participants. We obtained H-Index scores for all participants.

### Participant characteristics and H-index scores

Table 1 shows that over 75% were female, approximately 40% indicated having a clinical research specialty, half had a medical degree (MD or DO), and about half provided clinical care. Over 60% of participants were not NIH new investigators, over 60% reported they were not early-stage investigators, and half had greater than 50% dedicated research time (Table 1). Not shown in Table 1 was that participants were 90% White and non-Hispanic and had median H-Index scores of 13 (IQR = 7, 25) ranging from 0 to 94.

**Table 1.** Participant characteristics and mean scores on sections 1 and 2 of the Researcher Investment Tool

	Overall		Section 1: Researcher Experience		Section 2: Researcher Perceptions	
	*n*	%	Mean	*p*-value	Mean	*p*-value
Total	30	100%				
Sex^1^						
Male	6	20%	3.5		3.4	
Female	23	77%	2.9	0.057	2.9	0.254
Category of research specialty						
Clinical research	13	43%	3.0		3.2	
All other research specialties^2^	17	57%	3.1	0.716	2.8	0.259
Highest academic degree						
MD or DO	15	50%	3.1		3.2	
All other academic degrees^3^	15	50%	2.9	0.571	2.8	0.225
Providing clinical care						
Yes	16	53%	3.0		3.1	
No	14	47%	3.0	0.921	2.9	0.467
NIH New Investigator Status						
Yes	11	37%	2.8		2.6	
No	19	63%	3.1	0.328	3.2	0.033*
NIH Early-Stage Investigator Status^4^						
Yes	8	27%	2.9		3.0	
No	19	63%	3.2	0.165	3.0	0.836
Percent dedicated time for research						
Greater than 50%	15	50%	3.0	0.974	2.9	0.522
Less than or equal to 50%	15	50%	3.0		3.1	

*Notes:* NIH = National Institutes of Health; not reported - 90% of respondents were White and not Hispanic/Latinx; “ref” indicates the reference group.

^1^ Participants who indicated sex as “Prefer not to say” (*n* = 1) dropped from analyses.

^2^ All other research specialties included basic science research (*n* = 7), health services research (*n* = 2), community or public health research (*n* = 5), and other (*n* = 3).

^3^ All other academic degrees included PhD/ScD (*n* = 10), Master’s degree (*n* = 3), and other (*n* = 2).

^4^ Participants who indicated NIH Early-Stage Investigator Status as “Not sure” (*n* = 3) dropped from analyses.

* p < 0.05.

### RIT experience and perception scores and participant characteristics

There were no differences between participant demographics (gender, research specialty, academic degree, and provision of clinical care) and overall mean RIT scores for researcher experience (section one) and researcher perceptions of institutional support (section two; Table 1). Similarly, there were no differences between NIH status (new or early-stage investigator), and percent dedicated time for research and overall mean RIT scores for experience. This broad look at overall mean RIT scores within the experience and perceptions sections indicated that participants who were a NIH new investigator had a 0.65 point lower mean RIT score (lower perceived institutional support for research), compared to participants that were not NIH new investigators (*p* < 0.05; Table 1). The supplemental table includes mean scores on the RIT *domains* within section 1, by participant demographics and other characteristics.

### RIT domain scores

**Figure 2.**
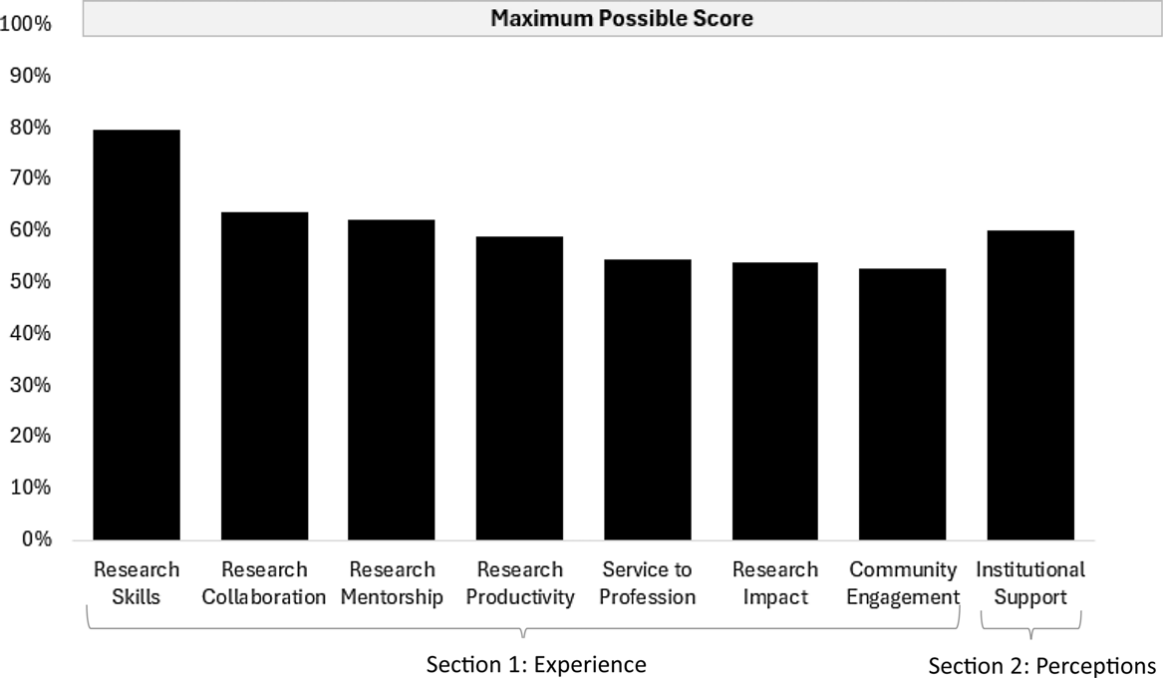
The percentage of total score out of the maximum score for each Researcher Investment Tool domain.

Table 2 shows mean RIT domain and sub-domain scores ranging from a high mean of 4.0 (sd = 0.7) for the research skills domain to a low mean of 2.6 for researcher productivity (sd = 0.7) and community engagement (sd = 1.1) domains. Figure 2 shows the percent of each domain score (mean/maximum) achieved in reference to 100%. Four domains in the Researcher Experience section (research productivity, service to the profession, research impact, and community engagement) fell below 60%, as did the one domain in the Researcher Perceptions of Institutional Support section.

**Table 2.** Maximum score, mean, standard deviation, and range for the Researcher Investment Tool

	Maximum Score	Mean	Standard Deviation	Range
**Section 1: Researcher Experiences (*n* = 30)**				
Total score	385	237.7	54.8	116–346
Item score	5	3.0	1.3	1–5
Domain 1: Research Skills				
Total score	60	47.7	8.7	24–60
Item score	5	4.0	0.9	1–5
Score of individual items in the domain				
Designing a research project	5	4.2	0.8	3–5
Writing a research proposal	5	4.1	0.7	3–5
Applying for research funding	5	3.8	1.0	2–5
Collaborating with other researchers	5	4.4	0.7	2–5
Leading a research team	5	3.7	1.1	2–5
Writing a research application for IRB or IACUC	5	3.9	0.8	2–5
Tracking the progress of a research study	5	3.8	1.0	1–5
Managing research data	5	4.0	1.0	2–5
Analyzing research data	5	4.0	1.0	2–5
Delivering a research presentation	5	4.3	0.8	2–5
Writing a research manuscript	5	4.2	0.9	2–5
Closing a research study	5	3.5	1.2	1–5
Domain 2: Service to Profession				
Total score	65	35.2	12.4	17–61
Item score	5	2.7	1.5	1–5
Score of individual items in the domain				
Leading a research facility or unit	5	2.4	1.4	1–5
Teaching research skills	5	3.4	1.1	2–5
Mentoring researchers	5	3.4	1.0	2–5
Serving as a reviewer for a research manuscript	5	3.8	1.0	2–5
Serving on an IRB/IACUC review team	5	1.7	1.2	1–5
Participating on a grant review team (any funder)	5	2.8	1.5	1–5
Participating in a NIH study section	5	2.2	1.5	1–5
Serving on an editorial board for a peer-reviewed scientific journal	5	2.0	1.5	1–5
Serving as a guest editor for a peer-reviewed scientific journal	5	2.0	1.4	1–5
Presenting your research locally, by invitation (e.g. invited to be a panelist/keynote)	5	3.5	1.2	1–5
Presenting your research nationally, by invitation (e.g. invited to be a panelist/keynote)	5	3.3	1.3	1–5
Presenting your research internationally, by invitation (e.g. invited to be a panelist/keynote)	5	2.5	1.4	1–5
Contributing to the development of new scientific guidelines	5	2.2	1.4	1–5
Domain 3: Research Productivity				
Total score	70	41.0	10.8	22–66
Item score	5	2.6	1.3	1–5
Score of individual items in the domain				
Receiving research funding from internal/institutional sources	5	3.3	0.8	2–5
Receiving research funding from external non-Federal source	5	3.0	1.1	1–5
Receiving research funding from industry sources	5	1.6	1.0	1–4
Receiving research funding from external Federal source (excluding NIH funding) (e.g., USDA)	5	2.4	1.3	1–5
Receiving research funding from NIH F grant (e.g., F31-F32)	5	1.4	1.0	1–5
Receiving research funding from NIH early-career K grant (e.g., K01, K08, K23, K24)	5	1.4	1.1	1–5
Receiving research funding from NIH R grant (e.g., RO3, R21, RO1)	5	2.1	1.5	1–5
Publishing a peer-reviewed manuscript	5	4.1	0.9	2–5
Publishing research in a high impact journal	5	3.3	1.1	1–5
Having research cited by other authors	5	3.7	0.9	2–5
Receiving inquiries from other researchers about your work	5	3.3	1.1	1–5
Translating research to the public using media (e.g., news articles, editorial, video interview)	5	2.6	1.3	1–5
Having research findings lead to changes in policies/procedures in health care (e.g., coding)	5	2.2	1.1	1–4
Having research findings influence best practices in care	5	2.2	1.1	1–4
Having research findings impact basic clinical and translational processes	5	2.1	1.0	1–4
Having research findings impact biomedical research progress	5	2.1	1.1	1–4
Domain 4: Research Collaboration				
Total score	60	38.1	10.2	15–57
Item score	5	3.2	1.2	1–5
Sub-Domain 1: Team Composition				
Total score	25	15.2	4.6	5–25
Item score	5	3.0	1.3	1–5
Score of individual items in the domain				
Participating in one or more multi-disciplinary research teams	5	3.7	1.1	1–5
Participating in one or more multi-institutional research teams	5	3.7	1.0	1–5
Leading multi-disciplinary research teams	5	2.8	1.2	1–5
Leading multi-institutional research teams	5	2.5	1.2	1–5
Participating in a research team that includes a patient voice	5	2.4	1.3	1–5
Sub-Domain 2: Team Collaboration				
Total score	35	22.9	6.3	10–34
Item score	5	3.3	1.2	1–5
Score of individual items in the domain				
Co-authoring research with a multi-disciplinary team	5	3.7	1.1	1–5
Co-authoring research with a multi-institutional team	5	3.7	1.1	1–5
Seeking out new partners (internal) to engage in research	5	3.4	0.9	2–5
Seeking out new partners (multi-institutional) to engage in research	5	3.3	1.1	2–5
Expanding research efforts to include cross-disciplinary approaches	5	3.1	1.1	1–5
Collaborating with multiple researchers on grant proposals (e.g., multi-PI, co-investigator)	5	3.1	1.1	1–5
Receiving multi-investigator grant funding	5	2.5	1.2	1–5
Domain 5: Research Mentorship				
Total score	75	46.4	13.5	18–72
Item score	5	3.1	1.3	1–5
Sub-Domain 3: Mentee Experience				
Total score	35	22.3	7.3	8–35
Item score	5	3.2	1.2	1–5
Score of individual items in the domain				
Receiving research mentorship (any type)	5	3.4	1.1	1–5
Meeting with mentor(s) on a routine basis	5	3.2	1.2	1–5
Creating a mentee/mentor plan	5	2.8	1.1	1–5
Being introduced to colleagues in the field by mentor(s)	5	3.2	1.1	1–5
Participating in research projects with mentor(s)	5	3.5	1.1	2–5
Gaining new skills from mentor(s)	5	3.4	1.1	1–5
Developing a research career plan with mentor(s)	5	2.8	1.4	1–5
Sub-Domain 4: Mentor Experience				
Total score	40	24.1	9.1	9–40
Item score	5	3.0	1.3	1–5
Score of individual items in the domain				
Mentoring students	5	3.8	1.0	2–5
Mentoring junior researchers	5	3.2	1.2	1–5
Assisting mentee(s) with their own grant proposals	5	2.7	1.3	1–5
Assisting mentee(s) with their own funded project	5	2.6	1.4	1–5
Assisting mentee(s) with writing	5	3.3	1.2	1–5
Advocating for compensated research time for mentee(s)	5	2.4	1.5	1–5
Introducing mentee(s) to new research opportunities	5	3.0	1.4	1–5
Introducing mentee(s) to colleagues in the field	5	3.1	1.2	1–5
Domain 6: Community Engagement				
Total score	25	13.1	5.5	5–24
Item score	5	2.6	1.2	1–5
Score of individual items in the domain				
Reaching out to engage community partners in my research activities	5	2.6	1.2	1–5
Identifying common research interests with the community	5	2.6	1.2	1–5
Aligning my research interests with community priorities	5	2.8	1.3	1–5
Participating in a research team that included a community voice	5	2.6	1.3	1–5
Communicating research findings back to community partners	5	2.5	1.1	1–4
Domain 7: Research Impact				
Total score	30	16.1	5.4	8-25
Item score	5	2.7	1.1	1–5
Score of individual items in the domain				
Participating in research that has positively influenced health outcomes	5	2.9	1.1	1–5
Participating in research that has positively influenced health policy	5	2.4	1.2	1–5
Participating in research that has positively influenced the biomedical field	5	2.4	1.3	1–5
Participating in research that has generated new theory or concepts	5	2.7	1.0	1–4
Participating in research that has been translated to clinical practice	5	2.6	1.1	1–4
Participating in research that has influenced the work of other researchers	5	3.1	1.0	1–5
**Section 2: Researcher Perceptions (*n* = 30)**				
Domain 8: Institutional Support				
Total score	55	32.9	8.9	17–53
Item score	5	3.0	1.1	1–5
Score of individual items in the domain				
Leadership in my organization values my research	5	3.2	0.9	2–5
My organization offers funding opportunities to support my research	5	2.9	1.1	1–5
My organization promotes multi-disciplinary research (mine or others)	5	3.0	1.1	1–5
My organization is committed to helping me build research skills	5	2.8	1.1	1–5
My organization recognizes my research accomplishments	5	2.9	1.0	1–5
My organization provides mentorship to support my career advancement	5	2.4	1.1	1–5
My organization supports community-engaged research efforts	5	2.8	1.0	1–5
I have been given designated time set aside for research	5	3.4	1.0	1–5
I have the support I need from my organization to do my research	5	3.1	1.0	1–5
I have the tools I need to do my research	5	3.2	0.9	1–5
I am clear on the research expectations, given my current role	5	3.3	1.2	1–5

*Notes:* IRB = Institutional Review Board; IACUC = Institutional Animal Care and Use Committee; NIH = National Institutes of Health; USDA = United States Department of Agriculture; PI = Principal Investigator.

#### Section I. Researcher experience

##### Domain 1: research skills

As shown in Table 2, this domain was notably higher than all other domains, yet five of 12 items had a mean score below the domain mean: applying for research funding, leading a research team, writing an Institutional Review Board (IRB/IACUC) application, and tracking or closing a research study. Most participants reported high levels of skills collaborating with other researchers and delivering a research presentation.

##### Domain 2: service to the profession

This domain had nearly the lowest mean score across research service-related items. Eight of 13 items scored below the domain mean including: serving on an IRB/IACUC review team, serving on an editorial board or as a guest editor for a journal, contributing to scientific guidelines, presenting research internationally, leading a research facility/unit, or participating in a NIH study section.

##### Domain 3: researcher productivity

This domain had one of the lowest mean scores across 16 items reflecting research funding and research dissemination. A few items with averages below the domain mean revealed there was limited experience with NIH funding targeting early career support including F grants, K grants and from funds secured through industry sources.

##### Domain 4: research collaboration

The mean score for this domain was at 3.2 with the team composition sub-domain mean score lower and the team collaboration sub-domain mean score higher. Items below the domain mean included *leading* research teams that were multi-disciplinary or multi-institutional and participating in a research team that includes a patient voice. Overall, four items in this domain received the highest mean score including: multi-disciplinary team participation and authorship as well as multi-institutional team participation and authorship.

##### Domain 5: research mentorship

The domain mean score was 3.1 with the sub-domain mean scores for mentee experience 0.1 points higher and mentor experience 0.1 points lower. Six items had a mean score below the domain mean including: introducing mentees to new research opportunities, creating a mentee/mentor plan, developing a research career plan with a mentor, assisting a mentee with their own grant proposals or their own funded project, and advocating for compensated research time for a mentee. The two items with the highest levels of experience were mentoring students and participating in research projects with mentors.

##### Domain 6: community engagement

This domain had the other lowest mean score and the fewest number of items. The item-level mean scores ranged from the lowest for experience communicating research findings back to community partners to the highest for experience aligning research interests with community priorities.

##### Domain 7: research impact

The mean domain score was also low and had six items. The highest mean level of experience was for participation in research that has positively influenced the work of other researchers. The lowest levels of experience were reported across two items including participation in research that positively influenced health outcomes and participation in research that positively influenced health policy.

#### Section II. Researcher perceptions

##### Domain 8: perceptions of institutional support

As shown in Table 2, this domain had a mean score in the middle of other domains. The items with the most favorable perceived institutional support were related to having designated time for research and the participants’ level of clarity on the research expectations given their role. Items that fell below the domain mean included: my organization offers mentorship to support career advancement and research funding, my organization is committed to helping me build research skills, my organization recognized my research accomplishments, and my organization supports community-engaged research efforts.

### Common indicators: H-index, investigator status, dedicated time

Table 3 shows results from regression analyses exploring associations between RIT domain mean scores and common indicators (objective two) including H-Index, investigator status, and dedicated research time. Participant H-Index score was associated with several research experience RIT domains including research skills, service to profession, research productivity and research collaboration (Table 3). For every 10-point increase in H-Index score, mean score for research skills (*p* = 0.004), research productivity (*p* = 0.002) and research collaboration (*p* = 0.021) increased by 0.2 points, and for service to profession, mean score increased by 0.3 points (*p* = 0.001). Table 3 also shows that mean scores for most RIT domains were lower for new or early-stage investigators when compared to mean domain scores for all other participants, but only two comparisons were significant. We found that NIH new investigators had 0.7 points lower mean scores in the researcher perceptions of institutional support domain than all other participants (*p* = 0.033), and NIH early-stage investigators had 0.5 points lower mean scores in the research skills domain than all other participants (*p* = 0.044). Finally, participants who indicated having more than 50% time dedicated for research scored 1.1 points lower on the community engagement domain than participants with less time dedicated to research (*p* = 0.005; Table 3).

**Table 3.** Associations between common indicators and Researcher Investment Tool domain mean scores

Independent Variables	Section 1: Researcher Experience														Section 2: Researcher Perceptions	
	Domain 1: Research Skills		Domain 2: Service to Profession		Domain 3: Research Productivity		Domain 4: Research Collaboration		Domain 5: Research Mentorship		Domain 6: Community Engagement		Domain 7: Research Impact		Domain 8: Institutional Support	
	Coeff	*P*-value	Coeff	*P*-value	Coeff	*P*-value	Coeff	*P*-value	Coeff	*P*-value	Coeff	*P*-value	Coeff	*P*-value	Coeff	P-value
H-Index (10 point increase)	0.20**	0.004	0.30***	0.001	0.20**	0.002	0.19*	0.021	0.11	0.232	−0.03	0.800	0.17	0.053	0.07	0.432
NIH New Investigator (Yes vs. No)	−0.33	0.232	−0.11	0.776	−0.24	0.366	−0.24	0.458	−0.25	0.469	−0.76	0.070	−0.17	0.625	−0.65*	0.033
NIH Early-Stage Investigator (Yes vs. No)	−0.52*	0.044	−0.73	0.064	−0.49	0.063	−0.26	0.464	−0.10	0.791	0.15	0.752	−0.09	0.816	0.00	0.995
Dedicated Research Time (> 50% vs. ≤ 50%)	0.41	0.126	0.17	0.635	−0.09	0.730	0.03	0.917	0.04	0.916	−1.09**	0.005	−0.08	0.817	−0.19	0.522

*Notes:* NIH = National Institutes of Health; CI = Confidence Interval; Coeff = Coefficient.

* p < 0.05.

** p < 0.01.

*** p < 0.001.

## Discussion

Our use of the RIT provides baseline data across eight domains assessing researchers’ experience and perceptions of institutional support within an IDeA CTR initiative. The study’s results suggest variability in levels of research experience across and within the RIT domains, with research skills rated the highest, and moderate levels of perceived institutional support, suggesting both strengths and areas for continued improvement in fostering research careers. This study’s results also support the expectation that researchers with higher bibliometric productivity as measured by the H-Index have more skills, service, productivity, and collaboration, while new and early-stage investigators show lower mean scores in most RIT domains. Overall, these results provide valuable insight into the current state of clinical and translational researchers in a historically underfunded region while also contributing to literature focused on measuring the effectiveness of programs like CTR networks.

As expected, there was variability in average item and domain scores, indicating participants had different levels of experience and perceptions. The fact that the research skills domain had the highest mean score overall suggested that our group of participants had high to moderate levels of experience with conceptualizing research, collaborating with others, working with data, and disseminating findings. Given our focus on investigators who were seeking research support or funding, we expected this group would have experience with the fundamental competencies needed to carry out a research project. In addition to the research skills domain, these baseline findings suggest moderate levels of experience with research collaboration and research mentorship. Other studies have shown that fostering collaborative opportunities and mentoring programs can influence the productivity of researchers, particularly those who are early-stage investigators [30,41–44]. Similarly, perceptions of institutional support were moderate, reflecting the culture, practices, and resources used by an organization to foster research. In general, participants reported relatively favorable support among leaders who valued research, as well as designated time to conduct research, available tools, and the necessary organizational support needed to foster research. They also reported having a clear understanding of research expectations, given their role.

The research productivity and community engagement domains had the lowest mean scores. While average item scores revealed participants had moderate levels of experience securing research funding from institutional or non-federal sources, they reported limited experience with NIH funding mechanisms aimed at early-stage investigators – a key outcome tracked by CTR Network awardees. Efforts to monitor progress in this area over time may be needed to better understand how investments and supportive efforts impact a researcher’s productivity as well as barriers they continue to face. Similarly, although the IDeA CTR Network awards emphasize community engagement, these findings revealed lower levels of aligning research with community interests, involving of the community in research, and including community members in the dissemination of findings. The challenges of community-based participatory research are well documented in the literature [45–47]. In our case, there may be several factors at play including the scope of the research, the readiness of researchers to engage the community, the use of available NNE-CTR resources to connect researchers with the community (e.g., community engagement research navigators), and the availability of funds or organizational support to involve the community, all opportunities for future research.

Results from objective two focusing on the analysis of the H-Index, investigator status, and dedicated research time in relation to RIT domains were mixed. We hoped to further validate the RIT by exploring the relationship between these constructs based on our theoretical expectations, and we were only able to partially do so. We expected the H-Index would be associated with RIT domains, as we anticipated that those who published more and whose work was cited more frequently would have higher levels of research experience. While we found that this was the case, only four of the eight RIT domains were associated with the H-Index. No statistical relationship was found with the domains focused on mentorship, community engagement, research impact, or perceptions of institutional support. More work is needed to understand if, and how, these domains are related to bibliometric indicators. Although most associations between new and early-stage investigator status and RIT domains were not significant, all but one were in the negative direction, as expected. While we used the NIH definitions to determine self-report investigator status, future validation studies may benefit from including other approaches for categorizing early-career researchers. Given existing efforts to support junior-level clinical and translation researchers, it will be important to continue exploring how career progression changes, and the individual nature of the RIT may prove useful in measuring these changes longitudinally. The finding that those with more than 50% dedicated research time had the lowest mean score on the RIT domain for community engagement suggests that those with more research time are not engaging with community as much. This was not expected, but it may have been due to faculty start-up funding, or the relatively new focus on community engaged research within more traditional medical research or lab-based research, or other unmeasured factors. The fact that no other associations were significant with dedicated research time warrants further investigation. It is possible this metric may not be as useful, or perhaps there are alternative approaches to capture dedicated research time. Despite the mixed results exploring our study’s second objective, the analyses build on our prior psychometric work with the RIT and may add further support for the use of the RIT in CTR evaluation.

This study includes several important limitations. First, although our baseline measures proved useful in capturing the current level of research experience and perceptions of institutional support, repeated measures will be needed to determine changes in this group over time, given the goal of research independence - a key focus of our evaluation. We plan to administer the RIT again, in two years. However, it is difficult to estimate the amount of time needed to see measurable changes using the RIT. Second, our participants were selected due to their small research funding awards and NNE-CTR research support. Since project awards have been in place since 2017, several awardees have had additional time to advance their research careers, post project funding. Our results provide a snapshot at one point in time, regardless of when project funds were awarded. Capturing baseline data prior to receiving a research project award may have been more informative, but may not have provided as much variability in scores. Third, most of our respondents did not identify as new or early-stage investigators. While several may have been new to CTR, our sample is skewed to those with more research experience, likely impacting the H-Index scores. More work is needed in this area to address the limitations of our sample.

Fourth, the RIT provides a comprehensive measure that is useful to evaluators but challenging given the self-report nature, length, and reoccurring administration over time. Additional efforts are needed to understand which domains are essential for repeated measures and the appropriate frequency for assessing change as well as which domains prove to be most useful in assessing research career progression. Finally, our efforts to further study the RIT by comparing domain scores with existing and commonly used research measures demonstrated mixed results, providing validation in limited areas only. There is currently no “gold standard” instrument we can use for criterion validity testing. Therefore, we selected indicators we theoretically expected to be associated with our tool, yet the chosen indicators have limitations. For example, the H-Index tends to favor those with more research experience. We also relied on free platforms (e.g., Google Scholar and ResearchGate versus Scopus) to identify the H-Index score, and we categorized dedicated research time based on an arbitrary cut-off (greater than 50%) we used in prior evaluation work. .

## Conclusions

The RIT is a novel approach to assessing researcher experiences and perceptions of institutional support within an IDeA CTR initiative. As an evaluation tool, the RIT can be used to identify the strengths of a research network, moving beyond traditional research productivity measures, as well as opportunities to identify areas where added supports could foster research career progression.

## Supporting information

10.1017/cts.2025.10125.sm001 Joly et al. supplementary material Joly et al. supplementary material

## Supplementary material

For supplementary material/s referred to in this article, please visit https://doi.org/10.1017/cts.2025.10125.
