# Supplementary material for: Using the Researcher Investment Tool to inform a clinical and translational research initiative
Source: J Clin Transl Sci. 2025 Sep 12;9(1):e213. doi: 10.1017/cts.2025.10125 (PMC12529636; doi:10.1017/cts.2025.10125)
Supplement: Joly et al. supplementary material [file S2059866125101258sup001.docx]

Supplemental Table 1. Mean scores on the Researcher Investment Tool (RIT) domains within the researchers’ experience section 1, by participant demographics and other characteristics.

|  | **Section 1: Researcher Experience** | | | | | | | | | | | | | | |
| --- | --- | --- | --- | --- | --- | --- | --- | --- | --- | --- | --- | --- | --- | --- | --- |
|  | **Domain 1: Research Skills** | | | **Domain 2: Service to Profession** | **Domain 3: Research Productivity** | | | **Domain 4: Research Collaboration** | **Domain 5: Research Mentorship** | | | **Domain 6: Community Engagement** | **Domain 7: Research Impact** | | |
| Sex^1^ |  | | |  |  | | |  |  | | |  |  | | |
| Male | | | 4.4 | 3.3 | | | 3.1 | 3.8 | | | 3.6 | 2.9 | | | 3.0 |
| Female | | | 3.9 | 2.6 | | | 2.4 | 3.0 | | | 3.0 | 2.5 | | | 2.6 |
| Category of research specialty |  | | |  |  | | |  |  | | |  |  | | |
| Clinical research | 3.7 | | | 2.6 | 2.5 | | | 3.2 | 3.1 | | | 2.8 | 2.7 | | |
| All other research specialties^2^ | 4.2 | | | 2.8 | 2.6 | | | 3.2 | 3.1 | | | 2.5 | 2.7 | | |
| Highest academic degree |  | | |  |  | | |  |  | | |  |  | | |
| MD or DO | 3.9 | | | 2.7 | 2.6 | | | 3.1 | 3.3 | | | 3.0 | 2.9 | | |
| All other academic degrees^3^ | 4.0 | | | 2.7 | 2.5 | | | 3.2 | 2.9 | | | 2.3 | 2.5 | | |
| Providing clinical care |  | | |  |  | | |  |  | | |  |  | | |
| Yes | 3.8 | | | 2.7 | 2.6 | | | 3.1 | 3.2 | | | 2.9 | 2.7 | | |
| No | 4.1 | | | 2.8 | 2.5 | | | 3.3 | 3.0 | | | 2.3 | 2.6 | | |
| NIH New Investigator Status |  | | |  |  | | |  |  | | |  |  | | |
| Yes | 3.8 | | | 2.6 | 2.4 | | | 3.0 | 2.9 | | | 2.1 | 2.6 | | |
| No | 4.1 | | | 2.7 | 2.7 | | | 3.3 | 3.2 | | | 2.9 | 2.7 | | |
| NIH Early-Stage Investigator Status^4^ |  | | |  |  | | |  |  | | |  |  | | |
| Yes | 3.7 | | | 2.3 | 2.3 | | | 3.1 | 3.1 | | | 2.8 | 2.7 | | |
| No | 4.2 | | | 3.0 | 2.8 | | | 3.3 | 3.2 | | | 2.7 | 2.8 | | |
| Percent dedicated time for research |  | | |  |  | | |  |  | | |  |  | | |
| Greater than 50% | 4.2 | | | 2.8 | 2.5 | | | 3.2 | 3.1 | | | 2.1 | 2.6 | | |
| Less than or equal to 50% | 3.8 | | | 2.6 | 2.6 | | | 3.2 | 3.1 | | | 3.2 | 2.7 | | |
| NOTES: NIH=National Institutes of Health; not reported - 90% of respondents were white and not Hispanic/Latinx; "ref" indicates the reference group | | | | | | | | | | | | | | | |
| ^1^ Participants who indicated sex as "Prefer not to say" (n=1) dropped from analyses | | | | | | | | | | | | | | | |
| ^2^ All other research specialties included basic science research (n=7), health services research (n=2), community or public health research (n=5), and other (n=3). | | | | | | | | | | | | | | | |
| ^3^ All other academic degrees included PhD/ScD (n=10), Master's degree (n=3), and other (n=2). | | | | | | | | | | | | | | | |
| ^4^ Participants who indicated NIH Early-Stage Investigator Status as "Not sure" (n=3) dropped from analyses | | | | | | | | | | | | | | | |
